# Statin Use Is Associated with Better Prognosis of Patients with Prostate Cancer after Definite Therapies: A Systematic Review and Meta-Analysis of Cohort Studies

**DOI:** 10.1155/2022/9275466

**Published:** 2022-11-15

**Authors:** Ye An, Jian-Xuan Sun, Meng-Yao Xu, Chen-Qian Liu, Jin-Zhou Xu, Xing-Yu Zhong, Jia Hu, Qi-Dong Xia, Heng-Long Hu, Shao-Gang Wang

**Affiliations:** Department and Institute of Urology, Tongji Hospital, Tongji Medical College, Huazhong University of Science and Technology, No. 1095 Jiefang Avenue, Wuhan 430030, China

## Abstract

**Objective:**

Although the prognostic effect of statins on patients with prostate cancer (PCa) has been frequently evaluated, a consistent result is still lacking. We aimed to evaluate the association between statin use and mortality among patients with PCa after definite therapies.

**Methods:**

A systematic search of PubMed and other databases for cohort studies about the effect of statins on patients with PCa was performed until April 2022. Meta-analysis was performed using R software version 4.1.2.

**Results:**

24 cohort studies involving 369, 206 participants were finally included. We found statin use significantly reduced the risk of prostate cancer-specific mortality (PCSM) with a pooled hazard ratio (pHR) = 0.76 (95% CI: 0.69–0.84, 18 studies), especially for postdiagnostic statin users: pHR = 0.81 (95% CI: 0.77–0.85) and patients who accepted androgen deprivation therapy (ADT): pHR = 0.69 (95% CI: 0.59–0.81). Statin use was also associated with a 24% reduction in the risk of all-cause mortality (ACM): pHR = 0.76 (95% CI: 0.68–0.85, 17 studies), especially for postdiagnostic statin users: pHR = 0.81 (95% CI: 0.78–0.85) and patients treated with ADT: pHR = 0.72 (95% CI: 0.63–0.82) or radiotherapy (RT): pHR = 0.68 (95% CI: 0.50–0.93).

**Conclusion:**

In conclusion, the use of statins could promote the prognosis of patients with PCa, especially for postdiagnostic users. For patients who received either ADT or radical prostatectomy (RP), statin use could decrease the PCSM. As for those who received either ADT or RT, statin use could decrease the ACM.

## 1. Introduction

Prostate cancer (PCa) is one of the most common malignant tumors; it has the second highest incidence and is the fifth leading cause of cancer-related death in men worldwide. Approximately 1.4 million incident cases were diagnosed, which led to more than 300 thousand deaths in 2020 [1]. Although localized PCa has a high 5-year survival rate, advanced PCa usually indicates a poor prognosis [2]. Radical prostatectomy (RP) and radiotherapy (RT) are the main treatments for localized PCa, and androgen deprivation therapy (ADT) is backbone of treatment for advanced PCa. Although ADT could slow tumor progression, a clinical state called castration-resistant PCa inevitably appears after treatment for a while. However, with a better understanding of PCa and the approval of multiple new drugs, the management of advanced PCa or castration-resistant PCa will change rapidly over the next decade [3].

Statins are a type of commonly used drug and are usually used to decrease serum cholesterol levels and prevent cardiovascular diseases by inhibiting cholesterol synthesis through suppression of HMG-CoA reductase. Beyond these effects, more and more evidence suggest that statins also play a role in the treatment of cancer, including colon cancer, breast cancer, and PCa [4–6]. Laboratory studies have proved that statins could limit cancer progression by promoting cell apoptosis, inflammation, and inhibition of cancer cell proliferation, adhesion, and angiogenesis [7–10]. Moreover, our previous study revealed that statins played a significant role in decreasing the risk of biomedical recurrence (BCR) in patients with PCa after definite therapies, especially RT [11].

Previous meta-analyses have demonstrated that statin use is associated with a reduced risk of prostate cancer-specific mortality (PCSM) and all-cause mortality (ACM) in PCa patients [12, 13]. But the number of cohort studies included in previous meta-analyses is limited, and many novel studies have been conducted since their publications, and the results of these new studies were inconsistent. Therefore, this systematic review and meta-analysis was conducted and aimed to reevaluate the association between statins and outcomes such as PCSM and ACM among men with PCa. Also, we conducted a subgroup analysis to examine the differences in prognosis among patients with different primary treatments or the time of statin initiation.

## 2. Methods

This study was conducted according to the Preferred Reporting Items for Systematic Reviews and Meta-Analyses (PRISMA) guidelines [14]. The protocol was registered on PROSPERO (ID: CRD 42022337522).

### 2.1. Literature Search

A systematic search of papers from Medline (PubMed), Embase (Ovid), and Cochrane was performed from inception to April 2022 by two independent reviewers (SJX and AY); conflicts were confirmed by the third reviewer XQD and finally resolved by consensus. All cohort studies evaluating the effect of statins on prognostic outcomes in patients with PCa were available with no language limitations. The literature was searched using the following terms: (“Prostatic Neoplasms” with its free words) and (“Statin” or “Atorvastatin” or “Cerivastatin” or “Compactin” or “Fluvastatin” or “HMG-CoA” or “Lovastatin” or “Mevastatin” or “Pravastatin” or “Rosuvastati'n or “Rosvastatin” or “Simvastatin”). The detailed search strategies of Medline (PubMed), Embase (Ovid), and Cochrane are shown in Supplement 1 Tables S1A–S1C. Also, potentially relevant studies were screened out from reference lists of articles retrieved, meta-analyses, and reviews.

### 2.2. Inclusion and Exclusion Criteria

Research articles were included if they satisfied the following criteria: (1) the study design was a cohort study; (2) studies examined the effect of statins on clinical outcomes in patients with prostate cancer; (3) the outcomes of interest were ACM or PCSM; and (4) relevant survival data with a hazard ratio (HR) estimate and its 95% confidence intervals (CIs) were reported. Studies satisfying the following criteria were excluded: (1) a case report, review, comment, or news item; (2) animal studies; (3) *in vitro* studies; and (4) studies with duration of follow-up shorter than 6 months.

### 2.3. Data Extraction and Quality Assessment

After exporting all retrieved articles to EndNote X9.3.3, duplicated articles were discarded. Two reviewers (SJX and AY) selected studies that met our criteria and then checked the results. Disagreements were resolved via discussion, involving the third reviewer (XQD). The following data were extracted from eligible articles: first author, year of publication, country of origin, study design, data sources, follow-up period, definition of statin use, tumor stage, primary treatment, adjustment variables, outcome, and HRs with corresponding 95% CIs. We extracted the risk estimate adjusted for the greatest number of confounding factors when a study provided more than one risk estimate.

We used the Newcastle–Ottawa scale (NOS) tool to evaluate the quality of studies, and the score of each study is presented in Supplement 2 Table S2. A study with a score of 7 or more was regarded as high quality.

### 2.4. Data Synthesis and Analysis

Heterogeneity across studies was measured by the *I*^2^ statistic and the 'Cochran's *Q*-test, with *I*^2^ > 50% and the *Q*-test*p* < 0.1 indicating significant heterogeneity [15]. The pooled hazard ratio (pHR) with corresponding 95% CIs for all included studies was obtained using a random effects model. Besides, the publication bias of included studies was examined using both Begg's [16] and Egger's [17] tests and then visualized as a contour-enhanced funnel plot. Where significant publication bias existed, the trim and fill method was carried out to normalize the publication bias [18], and the normalized combined effects will be used to verify the initial conclusion. We also performed meta-regression analysis to find the possible reasons responsible for heterogeneity, and we used the following parameters: publication year, median follow-up time, age, BMI value, Gleason score, PSA level, race, and tumor stage. Finally, subgroup analyses were performed stratified by primary treatment and the time of statin initiation.

We performed statistical analyses using R software version 4.1.2 and the package “meta.” A *P* value less than 0.05 indicated statistical significance.

## 3. Results

### 3.1. Study Characteristics

**Figure 1:**
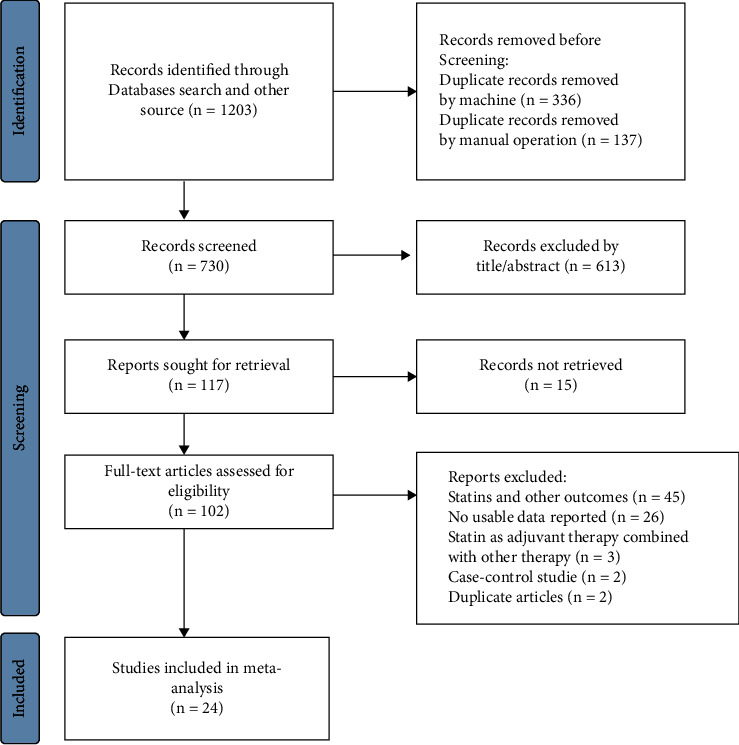
PRISMA flowchart for study selection.

A total of 1,203 citations were screened and assessed, and 24 cohort studies [19–42] were finally included in this study. The PRISMA flow diagram presented in Figure 1 shows the study selection process. Supplement 3 Table S3 shows the basic characteristics of the included studies. All the studies were published between 2010 and 2021, with at least 6 score of NOS results. Among these studies, 7 studies were conducted in the USA [20, 23, 24, 28, 37, 41, 42], 4 in Canada [21, 22, 29, 40], 4 in China [25, 26, 31, 36], 4 in Finland [19, 27, 32, 34], 1 in Italy [30], 1 in Denmark [33], 1 in Germany [35], 1 in the UK [38], and 1 in Norway [39]. 18 studies reported the association between statin use and PCMS, whereas 17 studies examined ACM.

### 3.2. Relationship between Statin Use and PCSM

**Figure 2:**
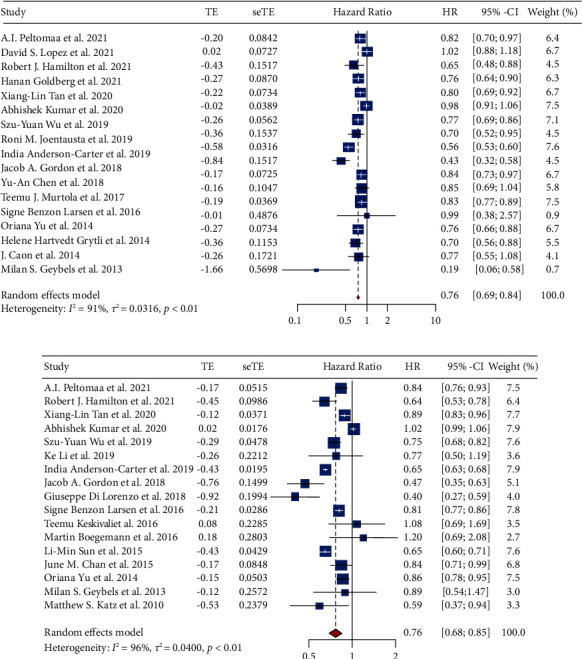
The effect of statins on PCSM or ACM of prostate cancer using a random effects model. (a) The forest plot for the HR of PCSM. (b) The forest plot for the HR of ACM.

**Figure 3:**
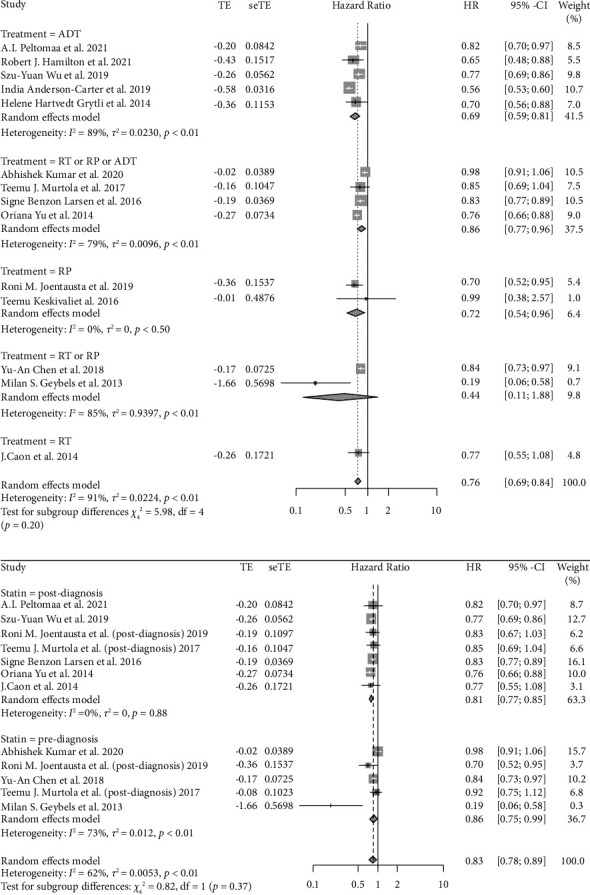
(a) The forest plot for the HR of PCSM with subgroup analysis by primary treatments. (b) The forest plot for the HR of PCSM with subgroup analysis by the initiation of statin use.

Eighteen studies with 347, 186 participants were included in the analysis of statin use and PCSM. The forest plot (Figure 2(a)) shows the overall effect of statin use on PCSM. The results suggested that statin use led to a significantly decreased risk of PCSM (pHR = 0.76, 95% CI: 0.69–0.84, *I*^2^ = 91%, random effects model). Subgroup analysis stratified by primary treatments is shown in Figure 3(a) and indicates that there is a significant reduction in PCSM among patients accepting ADT (pHR = 0.69, 95% CI: 0.59–0.81, *I*^2^ = 89%), RP (pHR = 0.72, 95% CI: 0.54–0.96, *I*^2^ = 94%), or RT or RP or ADT (pHR = 0.86, 95% CI: 0.77–0.96, *I*^2^ = 79%). Surprisingly, we found there was no statistical significance between PCSM and statin use when patients were treated with RT or RP. In the subgroup analysis stratified by the initiation of statin use (Figure 3(b)), we found there existed a significant reduction in PCSM among people accepting prediagnostic statin use (pHR = 0.86, 95% CI: 0.75–0.99, *I*^2^ = 73%) and postdiagnostic statin use (pHR = 0.81, 95% CI: 0.77–0.85, *I*^2^ = 0%).

As the heterogeneity of the main analysis and subgroup analysis was significantly high, we performed meta-regression. We constructed a univariate meta-regression model using the publication year, median follow-up time, age, BMI value, percentage of patients with a Gleason score ≥7, race, PSA level, and percentage of patients with a tumor stage ≥T3. We found tumor stage was significantly associated with PCSM (*P*=0.0237, see Supplement 4 Figure S1A), whereas other parameters were not significantly associated with PCSM.

**Figure 4:**
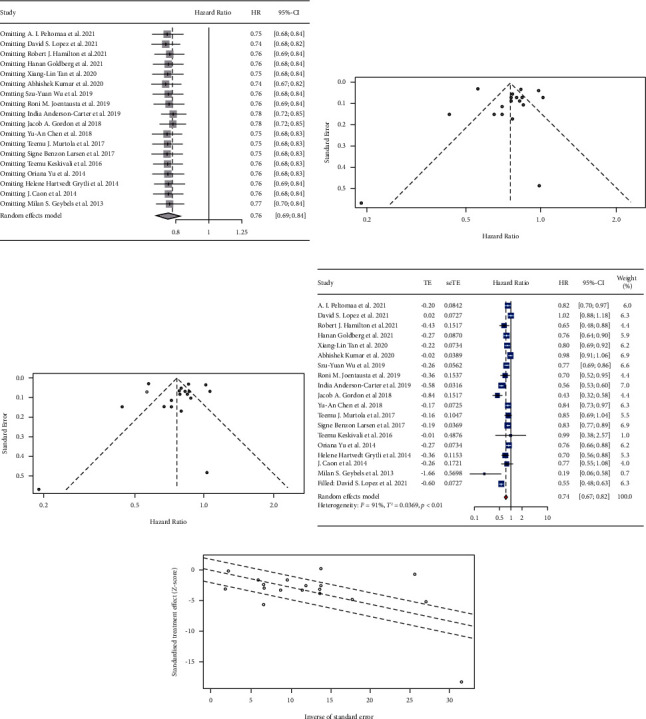
Sensitivity analysis and the detection of publication bias for included studies on the HR of the PCSM. (a) Sensitivity analysis by stepwise excluding the included studies. (b) The funnel plot. (c) The trim and fill funnel plot. (d) The filled forest plot. (e) The Galbraith plot. Effect sizes as *z*-scores plotted as a function of the inverse standard error for each study reported in the present study. The middle line is the line of best fit, while the upper and lower dashed lines represent the upper and lower 95% confidence limits.

Also, sensitivity analysis was performed to evaluate the effect of each study on the pHR. By stepwise excluding each study, we could observe that the overall estimates remained stable (Figure 4(a)). Both Begg's rank correlation test (*z* = −1.93, *P*=0.0534) and Egger's linear regression test (*t* = 0.02, *P*=0.9847) showed no evidence of significant publication bias. The contour-enhanced funnel plot showed a little asymmetry, as few studies were outside the dashed lines (Figure 4(b)). The trim and fill method estimated one study was missing due to publication bias (Figure 4(c)) and showed little evidence of publication bias. Then, we did a filled forest plot (Figure 4(d)), and the pHR was 0.74 (95% CI: 0.67–0.82, *I*^2^ = 91%, random effects model), which was consistent with our original result. The Galbraith plot showed a similar result, showing that most studies stood within the dashed lines (Figure 4(e)).

### 3.3. Relationship between Statin Use and ACM

**Figure 5:**
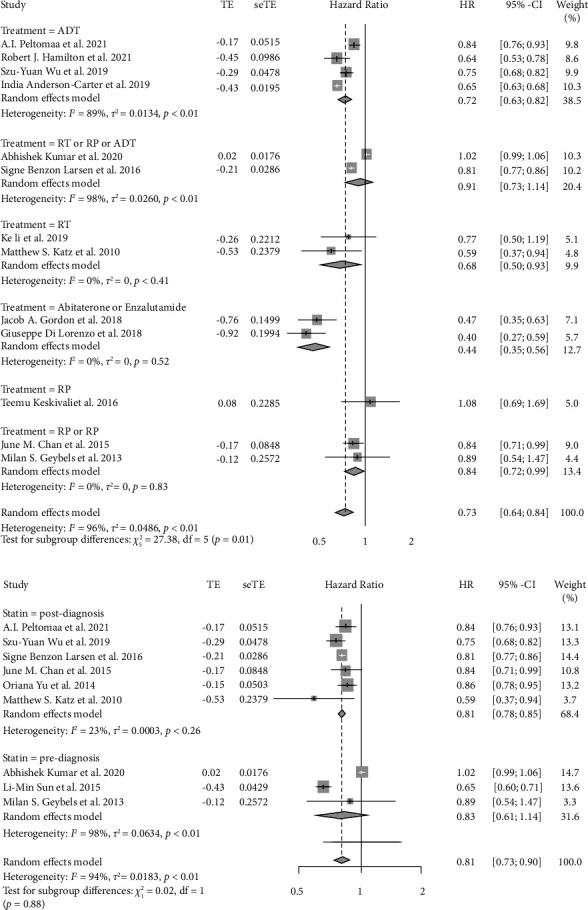
(a) The forest plot for the HR of ACM with subgroup analysis by primary treatments. (b) The forest plot for the HR of ACM with subgroup analysis by the initiation of statin use.

Seventeen studies with 246 and 167 participants were included in the analysis of statin use and ACM. As shown in the forest plot (Figure 2(b)), the result revealed a significant reduction in ACM among patients using statins (pHR = 0.76, 95% CI: 0.68–0.85, *I*^2^ = 96%, random effects model). In the subgroup analysis by primary treatment (Figure 5(a)), patients accepting ADT (pHR = 0.72, 95% CI: 0.63–0.82, *I*^2^ = 89%), RT (pHR = 0.68, 95% CI: 0.50–0.93, *I*^2^ = 0%), RT or RP (pHR = 0.84, 95% CI: 0.72–0.99, *I*^2^ = 0%), or abiraterone or enzalutamide (pHR = 0.44, 95% CI: 0.35–0.56, *I*^2^ = 0%) showed decreased risk of ACM, whereas the RP showed no effect on ACM. This result was not consistent with a previous study [43]. When stratified by the initiation of statin use (Figure 5(b)), only postdiagnostic statin use (pHR = 0.81, 95% CI: 0.78–0.85, *I*^2^ = 23%) was connected with a reduced risk of ACM.

A univariate meta-regression model was constructed using the parameters we mentioned above. We found the percentage of white people was associated with ACM (*P*=0.0021, see Supplement 4 Figure S1B), and other parameters were not associated with ACM.

Sensitivity analysis was presented in Supplement 4, Figure S2A, and the overall estimates remained stable after excluding each study. The contour-enhanced funnel plot (Supplement 4, Figure S2B) did not show good symmety, where some studies stood outside the dashed lines. However, both Begg's test (*z* = 0.25, *P*=0.8048) and Egger's test (*t* = −0.75, *P*=0.4647) showed no evidence of statistically significant publication bias. The trim and fill method was carried out, and it was estimated that two studies were missing due to publication bias (Supplement 4 Figure S2C). The filled forest plot (Supplement 4 Figure S2D) was carried out with pHR = 0.81 (95% CI: 0.70–0.93, *I*^2^ = 95%, random effects model), which indicated the reliability of our meta-analysis. The Galbraith plot also showed a similar result, showing that a few studies stood outside the dashed lines (Supplement 4 Figure S2E).

## 4. Discussion

This meta-analysis involving 24 studies with 369, 206 individuals reinvestigated the relationship between statin use and outcomes in patients with PCa and evaluated whether statin use contributed to different clinical outcomes when patients accepted different primary treatments. Our previous study has provided evidence that statins could reduce the risk of BCR in patients with PCa. However, the previous study focused on BCR and ignored other clinical outcomes; also, the study did not distinguish prediagnostic statin users from postdiagnostic users. Therefore, we conducted this meta-analysis to further evaluate the relationship between statins and clinical outcomes and instruct clinical medication. Our results revealed that statin use was associated with a significant reduction of PCSM and ACM. Subgroup analyses by primary treatment and initiation of statins were conducted. For PCSM, patients accepting ADT, RP, or RT or ADT, RP could benefit from statins. However, subgroups including ADT showed significant heterogeneity, which indicated individuals may not always benefit from statins when accepting ADT. Consistent with previous studies [13], our results demonstrated both postdiagnostic and prediagnostic statin users could obtain a reduced risk of PCSM. However, the prediagnostic statin use group showed high heterogeneity (*I*^2^ = 73%), indicating this result may not be suitable for all patients. As for ACM, patients accepting ADT, RT or RP, RT, abiraterone or enzalutamide showed potential benefits from statin use, where the ADT subgroup also had high heterogeneity. Although our study showed statin use did not reduce ACM for patients treated with RP, the number of studies in this subgroup was too limited, and further investigation was needed. In the subgroup analysis of ACM, we included two studies that used abiraterone or enzalutamide as the primary treatment. The result revealed statin use may reduce the risk of ACM when accepting abiraterone or enzalutamide, which was consistent with previous meta-analyses [44]. As the selection of primary treatment depends on certain subtypes of PCa, the patients involved in these two studies were all diagnosed with metastatic castration-resistant prostate cancer (mCRPC). Therefore, our study suggested that patients with mCRPC might benefit from statins when treated with abiraterone or enzalutamide. In 2016, an *in vitro* study discovered that statins could promote the therapeutic effect of enzalutamide in androgen-sensitive LNCaP and VCaP cells [45]. More randomized controlled trials and further studies are needed to clarify the effect of statins on enzalutamide use.

Additionally, we found only postdiagnostic statin use was associated with decreased risk of ACM but not prediagnosis. Alexandre et al. have pointed out [46] that prediagnostic statin users are more likely to be smokers, overweight, older, and have associated cardiovascular diseases or other diseases that might lead to a poor prognosis. Anyway, we observed a decreased risk of PCSM and ACM among patients accepting postdiagnostic statin use. Our findings could help to instruct clinical medication in patients with PCa.

Despite the fact that the antitumor effect of statins has been reported for years, many unknowns remain about their antitumor mechanisms, especially for PCa. It is known that statins can decrease cholesterol synthesis by suppressing HMG-CoA reductase. The presence of PCa was reported to be tightly related to cholesterol accumulation in prostatic tissues [47]. PCa could abnormally accumulate cholesterol by affecting the ABCA1 promoter [48] and activating the PI3K-AKT-mTOR signaling pathway [49]. Statins could suppress tumor growth by breaking the cholesterol balance in prostatic tissues. Cholesterol is a precursor for androgen synthesis, and androgen is essential for the initiation and progression of PCa. Therefore, it is not difficult to understand that statins could suppress androgen synthesis and improve the effect of ADT. This is in accordance with our results that statins could improve prognosis of patients with PCa when treated with ADT. Additionally, it was reported that statins competitively reduced the uptake of dehydroepiandrosterone sulfate, thus inhibiting the tumor's androgen synthesis [50].

However, there are limitations to this study. First, most included studies did not provide the baseline serum cholesterol levels. As statins are prescribed to decrease cholesterol levels, the serum cholesterol level might be a potential confounder in the analysis. Second, the definition of statin use varied among the included studies. The types of statins, doses of statins, initiation time of statin use, and duration of statin use were various or not complete in the included studies. The differences among these factors may lead to heterogeneity. Third, some patients received more than one kind of treatment, which could influence the result of subgroup analysis when stratified by primary treatment. Fourth, although most results of studies have been adjusted for important covariates, those unadjusted factors might have an impact on the results of individual studies.

## 5. Conclusion

In conclusion, the use of statins is beneficial for ACM and PCSM, especially for postdiagnostic users. For patients who received either ADT or RP, statin use could decrease the PCSM. As for those who accepted either ADT or RT, statin use could decrease ACM. However, for patients accepting ADT, statin use may not always be beneficial for them. In future studies, prospective studies or large-sample randomized controlled trials are needed to further elucidate the effects and specific mechanisms of statins in PCa.

## Data Availability

All the data analyzed in this study are included within article/supplementary material and are available from the corresponding authors upon request.

## Supplementary Materials

Supplementary Materials Supplement 1: Details of the search strategy to retrieve the studies. Supplement 2: Newcastle–Ottawa scale for assessing the quality of studies in meta-analysis. Supplement 3: Characteristics of included studies in the systematic review and meta-analysis. Supplement 4: Meta-regression and sensitivity analysis.
