# Supplementary material for: Statin Use Is Associated with Better Prognosis of Patients with Prostate Cancer after Definite Therapies: A Systematic Review and Meta-Analysis of Cohort Studies
Source: J Oncol. 2022 Nov 15;2022:9275466. doi: 10.1155/2022/9275466 (PMC9681552; doi:10.1155/2022/9275466)
Supplement: Supplementary Materials — Supplement 1: Details of the search strategy to retrieve the studies. Supplement 2: Newcastle–Ottawa scale for assessing the quality of studies in meta-analysis. Supplement 3: Characteristics of included studies in the systematic review and meta-analysis. Supplement 4: Meta-regression and sensitivity analysis. [file 9275466.f1.zip › Supplement 2.docx]

**Supplement 2 Table S2 Newcastle-Ottawa Scale for assessing the quality of studies in meta-analysis**

| Study | Selection | | | | Comparability | Outcome | | | Score |
| --- | --- | --- | --- | --- | --- | --- | --- | --- | --- |
|  | Representativeness of the  exposed cohort | Selection of the  nonexposed cohort | Ascertainment  of exposure | Demonstration that outcome of  interest was not present at start of  study | Comparability of cohorts on the  basis of the design or analysis | Assessment  of outcome | Was follow-up long enough  for outcomes to occur | Adequacy of  follow up of  cohorts |  |
| A. I. Peltomaa et al.  2021 | ✯ | ✯ | ✯ | ✯ | ✯ ✯ | ✯ | ✯ | ✯ | 9 |
| David S. Lopez et al.  2021 | ✯ | ✯ | ✯ | ✯ | ✯ |  | ✯ | ✯ | 7 |
| Robert J. Hamilton et al.  2021 | ✯ | ✯ |  | ✯ | ✯✯ | ✯ | ✯ | ✯ | 8 |
| Hanan Goldberg et al.  2021 | ✯ | ✯ | ✯ | ✯ | ✯ | ✯ | ✯ | ✯ | 8 |
| Xiang-Lin Tan et al.  2020 | ✯ | ✯ | ✯ | ✯ |  | ✯ | ✯ | ✯ | 7 |
| Abhishek Kumar et al.  2020 | ✯ | ✯ | ✯ | ✯ | ✯ ✯ | ✯ | ✯ | ✯ | 9 |
| Szu-Yuan Wu et al.  2019 | ✯ | ✯ | ✯ | ✯ | ✯ | ✯ | ✯ | ✯ | 8 |
| Ke li et al.  2019 | ✯ | ✯ |  | ✯ |  | ✯ | ✯ | ✯ | 6 |
| Roni M. Joentausta et al.  2019 |  | ✯ | ✯ | ✯ | ✯ | ✯ | ✯ | ✯ | 7 |
| India Anderson-Carter et al.  2019 | ✯ | ✯ | ✯ | ✯ |  | ✯ | ✯ | ✯ | 7 |
| Jacob A. Gordon et al.  2018 |  | ✯ |  | ✯ | ✯ | ✯ | ✯ | ✯ | 6 |
| Giuseppe Di Lorenzo et al.  2018 | ✯ | ✯ |  | ✯ | ✯✯ | ✯ | ✯ | ✯ | 8 |
| Yu-An Chen et al.  2018 |  | ✯ | ✯ | ✯ | ✯ | ✯ | ✯ | ✯ | 7 |
| Teemu J. Murtola et al.  2017 | ✯ | ✯ | ✯ | ✯ | ✯ | ✯ | ✯ | ✯ | 8 |
| Signe Benzon Larsen et al.  2017 | ✯ | ✯ | ✯ | ✯ | ✯ | ✯ | ✯ | ✯ | 8 |
| Teemu Keskivali et al.  2016 | ✯ | ✯ | ✯ | ✯ | ✯ | ✯ | ✯ | ✯ | 8 |
| Martin Boegemann et al.  2016 | ✯ | ✯ |  | ✯ | ✯ |  | ✯ | ✯ | 6 |
| Li-Min Sun et al.  2015 | ✯ | ✯ | ✯ | ✯ |  | ✯ | ✯ | ✯ | 7 |
| June M. Chan et al.  2015 | ✯ | ✯ |  | ✯ | ✯ | ✯ | ✯ | ✯ | 7 |
| Oriana Yu et al.  2014 | ✯ | ✯ | ✯ | ✯ | ✯ | ✯ | ✯ | ✯ | 8 |
| Helene Hartvedt Grytli et al.  2014 | ✯ | ✯ | ✯ | ✯ |  | ✯ | ✯ | ✯ | 7 |
| J. Caon et al.  2014 |  | ✯ | ✯ | ✯ |  | ✯ | ✯ | ✯ | 6 |
| Milan S. Geybels et al.  2013 | ✯ | ✯ | ✯ | ✯ | ✯ | ✯ | ✯ | ✯ | 8 |
| Matthew S. Katz et al.  2010 | ✯ | ✯ | ✯ | ✯ |  | ✯ | ✯ | ✯ | 7 |

**Note: A study can be awarded a maximum of one star for each numbered item within the Selection and Outcome categories. A maximum of two stars can be given for Comparability.**
