# Supplementary material for: Statin Use Is Associated with Better Prognosis of Patients with Prostate Cancer after Definite Therapies: A Systematic Review and Meta-Analysis of Cohort Studies
Source: J Oncol. 2022 Nov 15;2022:9275466. doi: 10.1155/2022/9275466 (PMC9681552; doi:10.1155/2022/9275466)
Supplement: Supplementary Materials — Supplement 1: Details of the search strategy to retrieve the studies. Supplement 2: Newcastle–Ottawa scale for assessing the quality of studies in meta-analysis. Supplement 3: Characteristics of included studies in the systematic review and meta-analysis. Supplement 4: Meta-regression and sensitivity analysis. [file 9275466.f1.zip › Supplement 3.docx]

**Supplement 3 Table S3 Characteristics of included studies in the systematic review and meta-analysis**

| **Study** | **Year** | **Country** | **Follow up period** | **Patient characteristics** | **Age (years), mean (s.d.) or median (IQR)** | **Gleason Score** | **Tumor stage** | **Primary treatment (s)** | **No. of patients** | **HR for PCSM** | **HR for ACM** | **Covariate adjustment** | **Newcastle-Ottawa Scale** |
| --- | --- | --- | --- | --- | --- | --- | --- | --- | --- | --- | --- | --- | --- |
| A. I. Peltomaa et al. | 2021 | Finland | 1996-2015, median 6.3 years | Finnish randomized study of screening for prostate cancer in the metropolitan areas of Helsinki or Tampere | (1) μ: 69.7; (2) μ: 69.0 | (1) ≥7: 63.5%; (2) ≥7: 66.1% | (1) T1-T2: 70.0%; T3-T4: 30.0%; (2) T1-T2: 60.8%; T3-T4: 39.2% | ADT | 4428 | HR: 0.82 (0.69 - 0.96) | HR: 0.84 (0.76 - 0.93) | Adjusted for age, tumor risk group, randomization group, use of other medication (antidiabetic and antihypertensive drugs, NSAIDs), and whether participants received radiation therapy in addition to ADT | 9 |
| David S. Lopez et al. | 2021 | USA | 2007-2015, mean 5.6 years | Using SEER-Medicare linked data we identified 89,346 NHW, NHB, and Hispanic men ≥ 65 years diagnosed with incident PCa (site recode 28,010) between 2007 and 2011 | (1) ≥70: 65.5%; (2) ≥70: 65.4% | (1) ≥8: 19.3%; (2) ≥8: 22.5% | (1) Localized: 93.2%; (2): Localized: 91.7% | testosterone replacement therapy (TTh) | 87574 | HR: 1.02 (0.88 - 1.17) | NR | Adjusted for age, race/ethnicity, Charlson Comorbidity Index (CCI), hypogonadism, hypertension, diabetes, use of insulin, muscular wasting and disuse atrophy, malaise and fatigue, osteoporosis, erectile dysfunction, depressive disorder, anterior pituitary disorder,  decreased libido, education (percentage of persons older than 25 years with less than 12 years education), percentage of adults below poverty line at census tract level, patients’ primary care (PCP), prostate-specific antigen (PSA), and mutual adjustment for TTh and statin，stage and grade at diagnosis | 7 |
| Robert J. Hamilton et al. | 2021 | Canada | 1999-2011， median follow-up of 6.9 yr | Histologically confirmed adenocarcinoma of the prostate after having completed definitive primary/salvage radiotherapy >12 mo prior to enrolment, a rising PSA level of ≥ 3 ng/ml above the postradiotherapy nadir, and no evidence of distant metastatic disease | (1) μ: 73.4 (51.4, 89.7); (2) μ: 74.9 (29.4, 89.3) | (1) ≥7: 78.2%; (2) ≥7: 73.9% | NR | ADT | 1364 | HR: 0.65 (0.48 - 0.87) | HR: 0.64 (0.53 - 0.78) | Adjusted for age, time from radiotherapy to ADT, baseline PSA, and prior ADT | 8 |
| Hanan Goldberg et al. | 2021 | Canada | 1994-2016, median 9.42yr | Ontario, men aged bigger than 66 yr with a history of a single negative prostate biopsy | (1) ≥70: 54%; (2) ≥70: 65.2% | NR | NR | NR | 5184 | HR: 0.76 (0.64 - 0.90) | NR | NR | 8 |
| Xiang-Lin Tan et al. | 2020 | USA | 2008-2014, median 42 months | Surveillance, Epidemiology, and End Results (SEER-18) database, patients with high-risk PCa being diagnosed from January 2008 to December 2011 | (1) ≥70: 51%; (2) ≥70: 47% | (1) ≥8: 35%; (2) ≥: 31% | (1) IIB: 70%; III: 10%; IV: 21%; (2) IIB: 64%; III: 10%; IV: 26% | RT or ADT | 10354 | HR: 0.80 (0.69 - 0.92) | HR: 0.89 (0.83 - 0.96) | Adjusted for cancer stage, ADT, radiation therapy, surgery, salvage radiation, secondary cancer therapy, propensity scores, and imbalanced variables after propensity scores adjustment | 7 |
| Abhishek Kumar et al. | 2020 | USA | 2000-2017, median 5.87 yr | Veterans Affairs Informatics and Computing Infrastructure to assemble a cohort of patients diagnosed with prostate cancer (PC) between 2000 and 2015 | (1)μ: 67.0 (7.5); (2)μ: 64.9 (8.5) | (1) ≥7: 59.5%; (2) ≥7: 59.5% | (1) T1: 70.4%; T2: 28.1%; T3: 2.5%; (2) T1: 68.1%; T2: 28.9%; T3: 3% | RT or RP or ADT | 68432 | HR: 0.98 (0.91 - 1.06) | HR: 1.02 (0.98 - 1.05) | NR | 9 |
| Szu-Yuan Wu et al. | 2019 | China | 2008-2014 | Taiwan National Health Insurance Research Data (NHIRD) linked to the TCR. The TCR was established in 1979 and contains 97% of the cancer cases in Taiwan | (1) μ: 73.23 (8.40); (2) μ: 73.36 (9.42) | NR | (1) T3-T4: 35%; N+: 65%; (2) T3-T4: 31%; N+: 69% | ADT | 5749 | HR: 0.77 (0.69 - 0.86) | HR: 0.75 (0.68 - 0.82) | Adjusted for cancer stage, cancer grade, year of the cancer diagnosis and the use of metformin, non-steroidal anti-inflammatory drugs and aspirin | 8 |
| Ke li et al. | 2019 | China | 2000-2010 | Males newly diagnosed with prostate cancer were identified from the NHIRD database (Taiwan National Health Insurance Research Data) | μ: 71.8 (8.7) | NR | NR | RT | 378 | NR | HR: 0.77 (0.50 - 1.19) | Adjusted for the baseline characteristics, including age, diabetes mellitus, hypertension, cardiovascular disease, peripheral artery disease, and atherosclerosis | 6 |
| Roni M. Joentausta et al. | 2019 | Finland | 1995-2015 | Cases were identified from national hospital discharge registry. Clinical data were amended from patient files of the treating hospitals. Information on co-morbidities, additional radiation- or chemotherapy, and causes of deaths were collected from national registries | (1) M: 63 (59, 66); (2) M: 61 (57, 66) | NR | NR | RP | 13876 | pre-diagnosis HR: 0.70 (0.52 - 0.95), post-diagnosis HR: 0.83 (0.67 - 1.03) | NR | Adjusted for age, multivariable adjusted model also for tumor extent, any use of chemotherapy or radiotherapy for PCa, diabetes, hypertension, coronary artery disease, and obesity | 7 |
| India Anderson-Carter et al. | 2019 | USA | 2000-2016 | National VA database was used to identify all men diagnosed with CaP who were treated with ADT for at least 6 months | (1) M: 73 (67, 78); (2) M: 76 (70, 81) | NR | NR | ADT | 87346 | HR: 0.56 (0.53 - 0.60) | HR: 0.65 (0.63 - 0.68) | Adjusted for age at ADT initiation, duration of ADT use, race, CCI, Agent Orange exposure, year of diagnosis, PSA at initiation of ADT, and Gleason score | 7 |
| Jacob A. Gordon et al. | 2018 | Canada | 2011-2016 | Medical records were reviewed at eight participating centers for patients with diagnosed mCRPC who were treated with second-line abiraterone or enzalutamide between January 2011 and January 2016 | (1) M: 74 (43, 94); (2) M: 72 (42, 96) | (1) ≥8: 54.8%; (2) ≥8: 57.6% | mCRPC | Abiraterone or Enzalutamide | 597 | HR: 0.43 (0.32 - 0.58) | HR: 0.47 (0.35 - 0.63) | NR | 6 |
| Giuseppe Di Lorenzo et al. | 2018 | Italy | 2010-2016 | The medical records of patients with documented mCRPC treated with abiraterone between September 2011 and August 2016 were reviewed at ten participating centers | (1) μ: 65.9 (6.3); (2) μ: 67.5 (6.2) | (1) ≥8: 25.4%; (2) ≥8: 50.5% | mCRPC | Abiraterone Acetate | 185 | NR | HR: 0.40 (0.27 - 0.59) | NR | 8 |
| Yu-An Chen et al. | 2018 | China | 1998-2010 | using the Taiwan National Health Insurance Research Database (NHIRD). A total of 15,264 PCa patients with hyperlipidemia records and medical claims from the Registry of Catastrophic illness were enrolled | (1) μ: 68.3 (7.8%); (2) μ: 68.3 (8.1%) | NR | NR | RT or RP | 3653 | HR: 0.84 (0.73 - 0.97) | NR | Adjusted for age, treatment of hormone therapy (including oral and injection), radical prostatectomy, radiotherapy, chemotherapy, and the comorbidities of diabetes, hypertension, stroke, cardiovascular disease and chronic obstructive pulmonary disease | 7 |
| Teemu J. Murtola et al. | 2017 | Finland | 1996-2012, median follow-up of 7.5 yr | 6537 prostate cancer cases diagnosed in the Finnish Randomized Study of Screening for Prostate Cancer population during 1996–2012 | (1) M: 67 (64–71); (2) M: 67 (63–71) | (1) ≥7: 33.2%; (2) ≥7: 35.8% | (1) Local: 93.5%; (2) Local: 89.6% | RT or RP or ADT | 6537 | post-diagnosis HR: 0.80 (0.65 - 0.98) pre-diagnosis HR: 0.92 (0.75 - 1.12) | NR | Adjusted for age, use of other drugs (aspirin, nonsteroidal anti-inflammatories, antidiabetic drugs, antihypertensive drugs, a-blockers, or 5a-reductase inhibitors) and for the screening trial arm and tumor stage, grade, and pre-diagnostic prostate-specific antigen. | 8 |
| Signe Benzon Larsen et al. | 2017 | Danish | 1998-2013, median 2.8 years (IQR, 1.3 to 5.1 years) | From nationwide Danish registries, we identified all patients with incident prostate adenocarcinoma from 1998 to 2011. Patients have no previous history of cancer, except nonmelanoma skin cancer | (1) ≥75: 30%; (2) ≥75: 33% | (1) ≥7: 67%; (2) ≥7: 57% | (1) Local: 60%; (2) Local: 55% | RT or RP or ADT | 31790 | HR: 0.83 (0.77 - 0.89) | HR: 0.81 (0.76 - 0.85) | Adjusted for age; calendar period; clinical stage; Gleason score; radical prostatectomy; use of aspirin, non-aspirin nonsteroidal anti-inflammatory drugs, antihypertensives, and other cardiovascular drugs; history of diabetes mellitus, chronic obstructive pulmonary disorder, ischemic heart disease, congestive heart disease, or moderate-to-severe kidney or liver diseases; and income, educational level, and marital status. | 8 |
| Teemu Keskivali et al. | 2016 | Finland | 1995-2013, median 8.6 years | Patients who underwent curative-intent radical prostatectomy at the Tampere University Hospital, Tampere, Finland during 1995–2009 | (1) M: 63; (2) M: 63 | (1) ≥7: 52.5%; (2) ≥7: 56.6% | (1) T1-T2: 99.2%; T3 and/or N1 0.8% (2) T1-T2: 98.4%; T3 and/or N1 1.6% | RP | 1314 | HR: 0.99 (0.38 - 2.57) | HR: 1.08 (0.69 - 1.69) | Adjusted for age, pathological TNM-stage, tumor Gleason score, surgical margin positivity, median total cholesterol and preoperative PSA | 8 |
| Martin Boegemann et al. | 2016 | Germany | 2010-2015; median 20.0 months (11.0–28.0) | Patients with mCRPC out of a prospectively maintained who were treated with Abiraterone at the Department of Urology, Muenster University Medical Center, between 02/2010-07/2015 | (1)M: 71.0 (67.5, 77.0); (2)M: 70.0 (62.0, 77.0) | (1) ≥8: 33.3%; (2) ≥8: 66.7% | mCRPC | Abiraterone Acetate | 108 | NR | HR: 1.20 (0.70 - 2.10) | Adjusted for status of metastases and line of therapy | 6 |
| Li-Min Sun et al. | 2015 | China | 1996-2011: average of 7.75 years | From the Taiwan National Health Insurance Research Database. The study cohort comprised 5179 patients diagnosed with prostate cancer who used statins for at least 6 months | (1) μ: 68.5 (8.14); (2) μ: 68.6 (8.76) | NR | NR | NR | 10358 | NR | HR: 0.65 (0.60 - 0.71) | Adjusted for age, sex, hormone therapy, treatment and comorbidities of diabetes, hypertension, stroke, CAD, and COPD | 7 |
| June M. Chan et al. | 2015 | USA | 1992-2010 | Patients diagnosed with localized prostate cancer (T3a or lower) from the Health Professionals Follow-Up Study between 1992 and 2008 and followed through 2010 | (1) μ: 70.7(6.8); (2) μ: 69.1(7.4) | (1) ≥7: 34%; (2) ≥7: 33% | (1) T1: 69%; T2: 29%; (2) T1: 64%; T2: 34% | RT or RP | 3949 | NR | HR: 0.84 (0.71 - 0.99) | Adjusted for age, time period, time from diagnosis to questionnaire, body mass index, vigorous physical activity, smoking, aspirin use, clinical stage, PSA at diagnosis, Gleason score, primary treatment, stroke, myocardial infarction, hypertension, and diabetes and comorbidities | 7 |
| Oriana Yu et al. | 2014 | Canada | 1998-2012, mean 4.4 years (SD=2.9) | Men newly diagnosed with nonmetastatic prostate cancer was identified using a large population-based electronic database from the United Kingdom | (1) μ: 71.9 (7.5); (2) μ: 71.1 (9.3) | (1) ≥7: 34.7%; (2) ≥7: 29.1% | NR | RT or RP or ADT | 11772 | HR: 0.76 (0.66 - 0.88) | HR: 0.86 (0.78 - 0.95) | Adjusted for age, year of prostate cancer diagnosis, ethnicity, excessive alcohol use, smoking status, obesity, chronic kidney disease, myocardial infarction, ischemic stroke, transient ischemic attack, peripheral artery disease, previous cancers, prostate-specific antigen level, Gleason score, metformin, sulfonylureas, thiazolidinediones, insulins, other oral anti-hypoglycemic agents, angiotensin-converting enzyme inhibitors, angiotensin receptor blockers, calcium channel blockers, β-blockers, diuretics, other antihypertensive drugs, aspirin, other nonsteroidal anti-inflammatory drugs, 5α-reductase inhibitors, pre-diagnostic statin use, prostate-specific antigen testing activity, prostatectomy, radiation therapy, chemotherapy, and androgen deprivation therapy | 8 |
| Helene Hartvedt Grytli et al. | 2014 | Norway | 2004-2011; median 39 mo | men reported to the Cancer Registry of Norway with a PCa diagnosis between 2004 and 2009 | NR | NR | NR | ADT | 3281 | HR: 0.70 (0.56 - 0.88) | NR | Adjusted for age, prostate-specific antigen level, Gleason score, clinical T stage, presence and type of metastases, performance status, and androgen-deprivation therapy initiated within 6 mo after diagnosis | 7 |
| J. Caon et al. | 2014 | Canada | median 8.4 years | Patients with prostate cancer treated with curative EBRT ± androgen deprivation therapy (ADT) | (1) M: 71; (2) M: 71 | NR | (1) Low risk 15%; intermediate 40% (2) Low risk 14%; intermediate 37% | RT | 2934 | HR: 0.769 (0.548 - 1.08) | NR | NR | 6 |
| Milan S. Geybels et al. | 2013 | USA | 2002-2011; median 7.6 years | Patients were identified via the Seattle-Puget Sound Surveil-lance, Epidemiology, and End Results (SEER) Program cancer registry | (1) μ: 63.1 (6.8); (2) μ: 60.9 (8.1) | (1) ≥7: 47.1%; (2) ≥7: 47.8% | (1) local: 72.3%; (2) Local: 72.6% | RT or RP | 1001 | HR: 0.19 (0.06 - 0.56) | HR: 0.89 (0.54 - 1.48) | Adjusted for age, Gleason score, stage at diagnosis, diagnostic PSA level, primary treatment approach, race, first-degree family history of PCa, body mass index, smoking status, lifetime alcohol consumption, regular aspirin use, regular non-aspirin NSAID use, history of diabetes mellitus, and history of PCa screening | 8 |
| Matthew S. Katz et al. | 2010 | USA | 1995-2006 median 4 (0–16) years | Men who underwent radical prostatectomy or radiotherapy for  prostate cancer between 1990 and 2003 identified in the Cancer of the Prostate Strategic Urologic Research Endeavor (CaPSURE) | μ: 64.4 (7.8) | NR | NR | RP or RT | 7042 | NR | HR: 0.59 (0.37 - 0.94) | Adjusted for clinical T stage and Gleason grade, initial PSA level and use of androgen-deprivation therapy (ADT), age, race, BMI, comorbid illness, and smoking status at diagnosis | 7 |

Abbreviation: PCSM, prostate cancer-specific mortality; ACM, all-cause mortality; RT, radiation therapy; RP, radical prostatectomy; ADT, androgen deprivation therapy; SD, standard deviation; IQR, inter quartile range; USA, United States of America; mCRPC, metastatic castration-resistant prostate cancer. (1) denotes statin users and (2) non-statin users.
