# Supplementary material for: Statin Use Is Associated with Better Prognosis of Patients with Prostate Cancer after Definite Therapies: A Systematic Review and Meta-Analysis of Cohort Studies
Source: J Oncol. 2022 Nov 15;2022:9275466. doi: 10.1155/2022/9275466 (PMC9681552; doi:10.1155/2022/9275466)
Supplement: Supplementary Materials — Supplement 1: Details of the search strategy to retrieve the studies. Supplement 2: Newcastle–Ottawa scale for assessing the quality of studies in meta-analysis. Supplement 3: Characteristics of included studies in the systematic review and meta-analysis. Supplement 4: Meta-regression and sensitivity analysis. [file 9275466.f1.zip › Supplement 4.docx]

**Supplement 4**


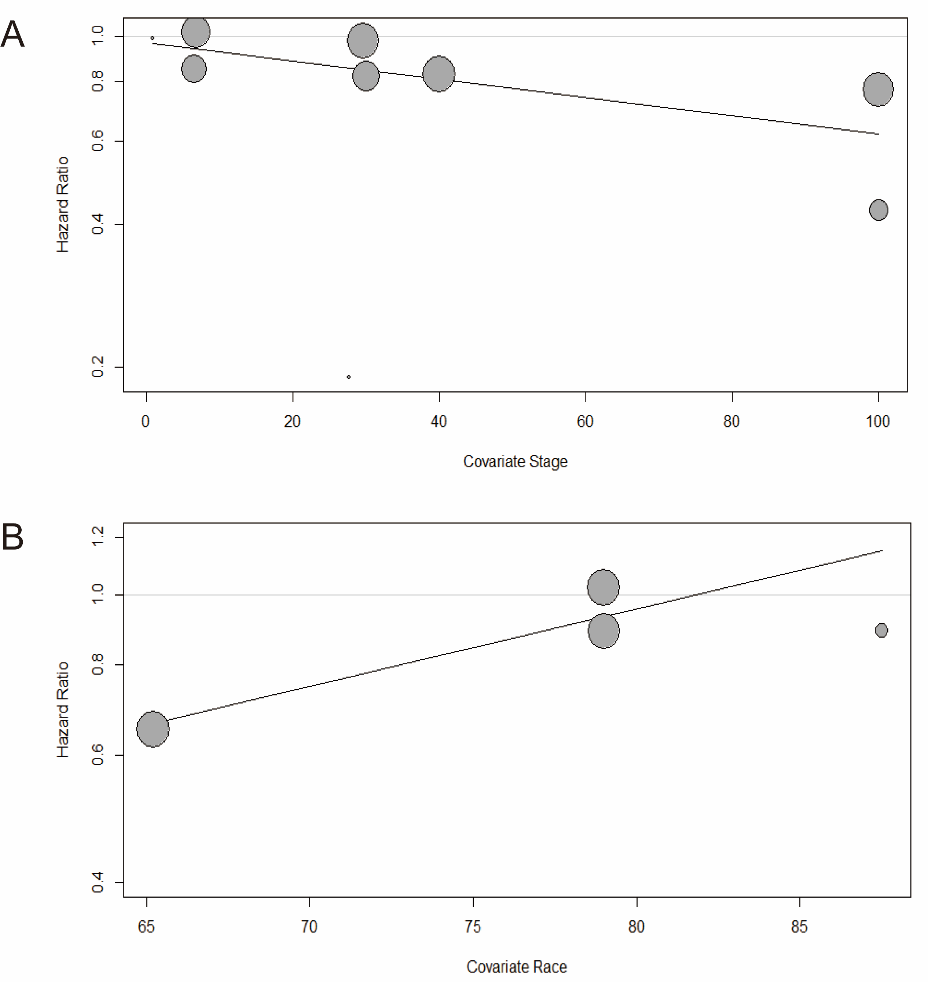


**Figure S1 The meta-regression results.**

**A** The meta-regression for HR of PCSM and tumor stage. **B** The meta-regression for HR of ACM and percentage of white people. Each dot represents an individual study. Symbol size represents sample size.


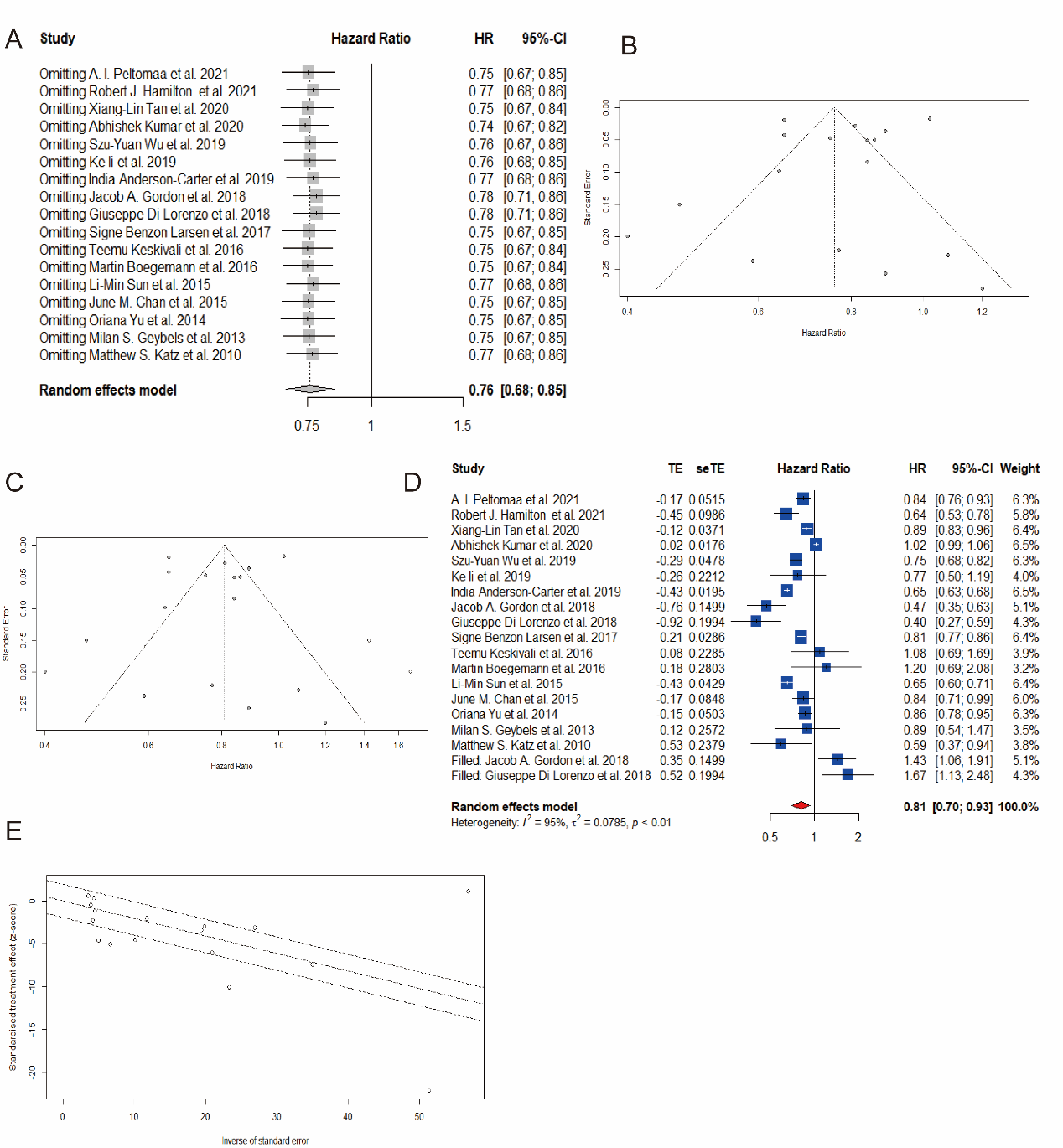


**Figure S2 Sensitivity analysis and the detection of publication bias for included studies on HR of ACM.**

**A** Sensitivity analysis by stepwise omitting the included studies. **B** The funnel plot. **C** The trim and fill funnel plot. **D** The filled forest plot. **E** The Galbraith plot. Effect size as z-scores plotted as a function of the inverse standard error for each study reported in the present study. The middle line is the line of best fit, while upper and lower dashed lines represent the upper and lower 95% confidence limits.
